# Coefficients of Thermal Expansion in Aligned Carbon Staple Fiber-Reinforced Polymers: Experimental Characterization with Numerical Investigation

**DOI:** 10.3390/polym17081088

**Published:** 2025-04-17

**Authors:** Julian Kupski, Lucian Zweifel, Miriam Preinfalck, Stephan Baz, Mohammad Hajikazemi, Christian Brauner

**Affiliations:** 1 Institute of Polymer Engineering, FHNW University of Applied Sciences and Arts Northwestern Switzerland, Klosterzelgstrasse 2, 5210 Windisch, Switzerlandlucian.zweifel@noriware.com (L.Z.);; 2 Deutsche Institute für Textil- und Faserforschung, Körschtalstrasse 26, 73770 Denkendorf, Germanystephan.baz@ditf.de (S.B.)

**Keywords:** carbon staple fibers, recycled composites, coefficients of thermal expansion (CTEs), fiber orientation distribution, tension-shear coupling, non-symmetric laminates

## Abstract

Carbon staple fiber composites are materials reinforced with discrete-length carbon fibers processed using traditional textile technologies, offering moderate mechanical properties and flexibility in manufacturing. These composites can be produced from recycled carbon staple fibers, aligned into yarn and tape-like structures, providing a more sustainable alternative while balancing performance, cost-effectiveness, and environmental impact. Aligning staple fibers into tape-like structures enables similar applications to those of continuous-fiber-based products, while allowing control over fiber orientation distribution, fiber volume fraction, and length distribution, which are all critical factors influencing both mechanical and thermo-mechanical properties. This study focuses on the experimental characterization and numerical investigation of Coefficients of Thermal Expansion (CTEs) in aligned carbon staple fiber composites. The effects of fiber orientation and volume fraction on coefficients of thermal expansion under different fiber alignment parameters are analyzed, revealing distinct thermal expansion behavior compared to typical aligned unidirectional continuous carbon fiber composite laminates. Unlike continuous unidirectional laminates, which typically exhibit transversely isotropic behavior without tensile–shear coupling, staple fiber composites demonstrate different in-plane axial, transverse, and out-of-plane CTE characteristics. To explain these deviations, a modeling approach is introduced, incorporating detailed experimental information on fiber distributions and microstructural features rather than averaged fiber orientation values. This involves a multi-scale analysis based on a laminate analogy through which all composite thermo-elastic properties can be predicted, accounting for variations in fiber orientations, volume fractions, and tape thicknesses. It is shown that while the local variation of fiber volume fraction has a small effect on the homogenized value of the coefficients of thermal expansion, fiber misalignment, tape thickness, and asymmetry in fiber orientation distribution will significantly affect the measurements of CTEs. For the case of carbon staple fiber composites, the asymmetry in fiber orientation distribution significantly influences the measurements of axial CTE. Fiber orientation asymmetry causes tensile–shear coupling under mechanical and thermal loading, leading to an unbalanced laminate with in-plane shear–tensile deformation. This coupling disrupts uniform displacement, complicating strain measurements and the determination of composite properties.

## 1. Introduction

Fiber-reinforced composites, particularly those incorporating Carbon Fibers (CFs), have gained widespread attention due to their exceptional mechanical properties, including high strength, stiffness, and durability [1]. These materials are increasingly used across industries such as aerospace, automotive, and renewable energy, where their high strength-to-weight ratios are especially valued. However, the growing demand for Carbon-Fiber-Reinforced Polymers (CFRPs) has also led to a significant accumulation of end-of-life composite materials, presenting environmental and economic challenges [2]. Current disposal methods, such as landfilling or incineration, are unsustainable, and the limited reusability of CFs exacerbates these issues [3]. While extensive research has focused on recycling methods, such as mechanical, chemical, and thermal reclamation techniques [4], these processes often compromise the structural integrity of the fibers, reducing their alignment and mechanical properties [5]. The composite industry faces increasing pressure to implement viable recycling strategies due to evolving waste management legislation and sustainability demands. Traditional recycling methods, such as mechanical and pyrolysis-based techniques, often compromise fiber properties, limiting their reuse in high-performance applications. Recent advancements in chemical recycling, particularly solvolysis in sub- and supercritical conditions, offer promising solutions by recovering long fibers with minimal degradation, enabling their reuse in new composites [6,7]. However, scaling up these processes remains a challenge, requiring further research on thermodynamic and mass transfer properties to optimize industrial feasibility [6,7]. Additionally, innovative approaches, such as fiber alignment techniques [8] and the development of degradable thermosets and low-viscosity thermoplastics, present opportunities for enhancing the sustainability and circularity of composite materials [9] in industries like aerospace, automotive, and wind energy.

After the fiber recovery process, the fibers can be processed through various techniques, including injection molding with short fibers [10] and sheet-molding, or bulk-molding compounds using short and medium-length fibers without specific orientation. These processes typically result in materials with low mechanical properties [11]. However, the highest potential for harnessing the mechanical performance of recycled Carbon Fibers (rCF) can be achieved when resulting carbon staple fibers after recycling are aligned in the form of yarn or tape-like structures using specific processes forming staple fiber-reinforced polymer composites [12,13]. It is noted that in comparison to other recycled fiber composites, such as those reinforced with glass or natural fibers, carbon fiber composites offer superior mechanical and thermal performance. While recycled glass fibers tend to suffer from reduced mechanical integrity and are often discontinuous and less thermally stable, natural fibers introduce moisture sensitivity and inconsistent thermal behavior. As highlighted in a comprehensive review [14], recycled carbon fibers demonstrate higher retention of mechanical properties and better compatibility with thermoset and thermoplastic matrices, positioning them as a more robust option for applications demanding thermal stability.

Carbon staple fiber composites consist of discontinuous fibers, typically ranging from a few millimeters to several centimeters in length, most of which aligned into yarn or tape-like structures. While fibers at the shorter end of this range may be too short to form yarns or tapes, they are often present alongside longer fibers [15]. The possibility of using recycled carbon fibers in tape manufacturing makes composites out of staple fibers an interesting material system to benefit CFRPs with a more circular approach. Unlike traditional short-fiber or non-woven, highly aligned staple fiber reinforcements result in composites with enhanced mechanical properties through their unique architecture, where fiber alignment and interlocking mechanisms facilitate a more uniform stress transfer and energy absorption. The use of highly aligned reinforcements addresses some of the limitations of discontinuous fiber composites [16], offering improved tensile strength, stiffness, and impact resistance. The processing of rCFs into composite tapes involves several methods, each contributing to the fiber’s alignment and dispersion, which is crucial for optimizing the mechanical properties of the final product.

In the wet-laying process [17], rCFs or a combination of rCFs and thermoplastic fibers, are mixed with a liquid to achieve a uniform dispersion of fibers. The mixture is pumped onto a sieve, where the fibers are filtered from the suspension. The fiber mats are then dried and optionally provided with a binder. The orientation of the fibers can be controlled by adjusting the movement of the sieve or the flow geometry of the headbox. This approach allows for only partial alignment of fibers, leading to slight improvement of thermo-mechanical properties in the composite [18].

The carding process, often used in non-woven fabric production, is designed to disentangle, blend, and align staple fibers to create a uniform web or sliver. During this process, the fiber flocks/bundles are opened into individual fibers and mixed thoroughly to ensure a uniform distribution. The fibers are passed onto the working elements of the carding machine, which are covered with metallic wires (like the teeth of a comb) that continuously engage with the fibers. As the fibers pass through this section, the action of the pins on the main cylinder straightens and untangles fiber bundles, using worker–stripper pairs to further separate and align them. As the fibers are worked on by the various rolls, they form a thin layer or web on the main cylinder. After the fibers have been processed and are aligned, the web is removed from the main cylinder by the doffer and gathered into a card web that can be stacked and densified to a non-woven form or compacted in the form of a single layer into a sliver. The sliver can then be further processed into yarns or tapes through additional stretching. The yarns can be compacted by twisting (e.g., ring spinning) or wrapping with a spiral covering filament yarn, while the tapes are consolidated by the incorporation of a binder. Both types of semifinished products can be used directly to produce composite components or processed into non-crimped, braided, or woven fabrics for further applications. In summary, the production of staple fiber tapes for composite application requires careful control over fiber alignment, length, and blend to achieve high-performance materials. Several investigations have explored the production of semi-finished products in tape form using recycled carbon fibers. The interested readers are referred to in Refs. [2,15,19,20,21] to find more information about different manufacturing techniques. The current work is concerned with carbon staple fiber composites that are manufactured using the roller carding process and subsequent drafting processes.

Despite the various alignment techniques and manufacturing processes employed for staple fiber composites, the resulting fiber alignment, distribution uniformity, fiber length, and volume fraction typically do not match the precision achieved in unidirectional (UD) continuous carbon fiber composites. Carbon staple fiber processing leads to a more heterogeneous fiber distribution. As a result, microstructural details in carbon staple fiber composites tend to exhibit greater variability, which affects the mechanical and thermal properties. To better understand and predict the behavior of staple fiber composites, it is crucial to obtain detailed insights into their microstructural characteristics. Advanced techniques such as X-ray Computed Tomography (CT), Scanning Electron Microscopy (SEM), and image analysis can provide useful data on fiber orientation tensors, fiber length distributions, and fiber volume fractions, enabling more accurate modeling and optimization of these materials. Recently, Zweifel et al. [12] have performed a multi-scale characterization on the microstructural organization of carbon staple fiber-reinforced polymer composites. They employed methods ranging from microscale to macroscale, such as image analysis and X-ray computed tomography to quantify the microstructural organizations resulting from different alignment processing methods. These results were compared with the results of mechanical tests to understand the relationship between fiber alignment and mechanical properties. The results show a significant influence of alignment on fiber orientation distribution, fiber volume fraction, tortuosity, and mechanical properties. Furthermore, different characteristics of the staple fiber tapes were identified and attributed to kinematic effects during movement of the sliver alignment unit, resulting in varying tape thicknesses and fuzzy surfaces.

Research on the characterization of aligned staple fiber composites is still in its infancy, requiring much deeper investigation to understand the relationships between manufacturing processes, fiber orientation distribution, and thermo-mechanical properties. To enable the use of these material systems in load-bearing structures, it is essential to develop a comprehensive understanding of their behavior under multi-axial thermo-mechanical loading. Such insights will enhance modeling techniques needed to predict the performance of highly aligned staple fiber composites. Current approaches to predicting the elastic properties of staple fiber composites are limited [22], focusing primarily on the axial in-plane Young’s modulus using simplified extensions of the rule of mixtures [12,23,24]. As will be discussed later in the modeling section, a more sophisticated approach is required to incorporate realistic physical effects of microstructural details on all laminate thermo-elastic properties. Additionally, investigating the in-plane transverse and out-of-plane properties of aligned staple fiber composites is critical, though the latter poses significant experimental challenges. This study aims to experimentally characterize the in-plane axial, in-plane transverse, and out-of-plane thermal expansion coefficients of highly aligned staple fiber-reinforced composites made of carbon fibers and epoxy resin. Two alignment techniques, based on the use of roller carding and subsequent drafting processes, were employed to create samples with fibers predominantly oriented at 0 degrees. Samples were prepared following standard procedures and tested using Thermo-Mechanical Analysis (TMA) to measure their Coefficients of Thermal Expansion (CTEs). The study sought to determine whether highly aligned staple fiber composites exhibit transverse isotropy, akin to typical continuous fiber unidirectional (UD) laminates. Distinct thermal expansion behavior was observed, prompting a detailed fiber orientation analysis using CT scans to generate orientation histograms. These data were incorporated into a new modeling approach that combines laminate analogy with accurate micromechanical formulations, accounting for fiber orientation details, variations in fiber volume fraction, tape thickness, and the transverse isotropic properties of carbon staple fibers. The different thermal expansion behavior of aligned staple fiber-reinforced laminates was investigated with regard to potential tensile–shear coupling effects arising from asymmetric fiber distribution.

Besides the primary challenges in recycled composites, factors like aging [25] and interfacial properties after recycling also warrant attention [26]. Moisture absorption, thermal cycling, and oxidation, which may occur during a composite part’s lifetime or the recycling process, can degrade fiber–matrix adhesion and fiber properties over time, affecting the mechanical performance of the resulting recycled composite. It is shown that surface treatments, such as oxidation and resizing with carbon nanotubes, can enhance interfacial adhesion in carbon fiber-reinforced polyamide 6 composites, improving tensile, flexural, and interlaminar shear strength [27]. Similarly, environmental factors like water immersion can weaken bond strength in fiber-reinforced composites, shifting failure modes and reducing fracture energy, as observed in CFRP-to-concrete bonded joints [28]. While this paper focuses on the CTE of carbon staple fiber composites, understanding the behavior of recycled composites against long-term environmental effects represents another important field of research.

## 2. Materials, Samples, and Microstructural Details

### 2.1. Carbon Staple Fiber Tapes

**Figure 1:**
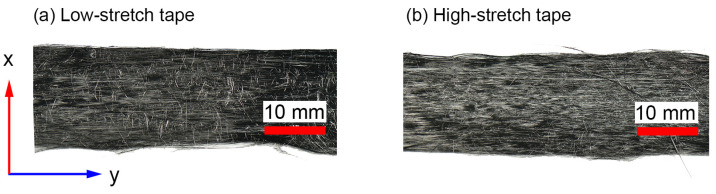
Top-view images of the tapes utilized in this study [12] depicting (**a**) low-stretch tape and (**b**) high-stretch tape, both with a nominal tape width of 16 mm.

The investigation will be performed using two types of aligned staple fiber tapes produced with varying alignment processing parameters, designated as low-stretch (LS) and high-stretch (HS). The average length (L) of a single fiber in these tapes was approximately 50 mm, and the average fiber diameter (d) was 7 μm. It is worth noting that for such a high aspect ratio (L/d) of the carbon fibers, the effects of fiber length on thermo-mechanical properties are negligible [29]. The fiber used in all samples was of the standard modulus/high-strength type, T700S-C-12K-50C, following the naming convention of Toray Industries, Inc. (Tokyo, Japan). Alongside the staple fiber tapes, a virgin reference plate was produced using a UD carbon fabric with 80 g/m^2^ using the same T700S-C-12K-50C tows. The epoxy resin system EPIKOTE^TM^ 05545/EPIKURE^TM^ 778/EPIKURE^TM^ 120 (Hexion, Columbus, OH, USA) was taken in this investigation, with Marbocote HP7 used as a release agent. Figure 1 shows optical images of both carbon staple fiber tape configurations utilized in this study. It is noted that these tapes are the same as those analyzed in the authors’ previous paper [12], focusing on fiber orientation distribution and axial elastic properties.

### 2.2. Specimen Manufacturing

The process of mixing the epoxy system was carried out using a Speedmixer DAC 1100.1 FVZ (Hauschild, Hamm, Germany), with two rounds of 2 min mixing intervals and reheating the resin to 80 °C between each round. Thorough mixing was achieved with a mixing speed of 1200 rpm while the resin was in its heated state. The resin was then degassed under a pressure of 100–200 mbar in a vacuum oven at 80 °C for 5–7 min to ensure the removal of any entrapped air. Table 1 summarizes the resin mixing method for the epoxy system. The mechanical properties of the fiber and matrix will be provided in the “results and discussions” section.

**Figure 2:**
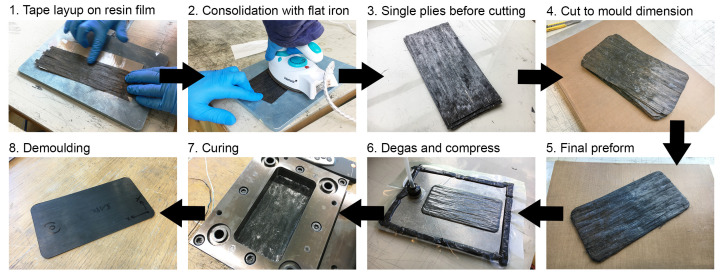
Manufacturing steps of flat test specimens in compression molding process [12].

A total of 12 layers of carbon staple fiber tapes were laminated to form flat test specimens using a compression molding tool with dimensions of 170 mm × 85 mm, as depicted in Figure 2. Resin films with a thickness of 55 μm and a weight of 60 g/m^2^ were produced using a hot melt laboratory coater and laminator, specifically the HLCL-1000 model from ChemInstruments (West Chester Township, OH, USA). These resin films were then used to manufacture a prepreg by film-stacking them with carbon fiber tapes in the desired layup configuration. The carbon fiber tapes were carefully aligned in parallel. Subsequently, each ply section was gently compressed using a hand-held flat iron. The iron surface was coated with a polytetrafluoroethylene film from Hightechflon GmbH & Co. KG (Konstanz, Germany) to prevent adherence to the resin film. A total of 12 plies were accurately cut to match the dimensions of the mold cavity. These plies were then stacked and subjected to vacuum degassing to ensure proper pre-consolidation before the curing process. The preform was inserted into the female part of the mold and subjected to a press curing cycle, which included a 60 min hold at 100 °C, followed by a ramp up to 140 °C and a second hold at 140 °C for another 60 min.

After manufacturing, the samples were mechanically tested, and the results are reported in Ref. [12]. Further fiber volume content measurements were performed, yielding average values of 55% for the virgin laminate, 46.3% for the highly stretched laminate (HS), and 39.5% for the low-stretched laminate (LS). These FVC values were derived visually using 2D images and 3D CT scans rather than through a muffle test.

### 2.3. Fiber Orientation Distribution

In a recent work by the authors [12], a detailed study was performed to obtain the microstructural details from samples manufactured above. To analyze the microstructural features of carbon staple fiber composites, a multi-scale characterization approach was employed, combining micro-level 3D fiber reconstruction and meso-level Fiber Orientation Distribution (FOD) analysis. High-Resolution (HR) and Ultra-High-Resolution (UHR) tomographic imaging were conducted on samples extracted from LS and HS plates. HR scans achieved a voxel resolution of 1 μm, while UHR scans provided finer detail with a resolution of 0.4 μm. Data processing was performed using Avizo 3D software (Version 2022.1; Thermo Fischer, Waltham, MA, USA) with the XFiber extension module to reconstruct individual fibers and compute key descriptors such as fiber Volume Fraction (VF) and FOD, representing fiber alignment statistically across the scanned regions. Regions of Interest (ROIs) within the composite were analyzed after applying median filtering to reduce noise. Fiber tracking and segmentation allowed for accurate extraction of descriptors, which were averaged across ROIs for statistical analysis.

**Figure 3:**
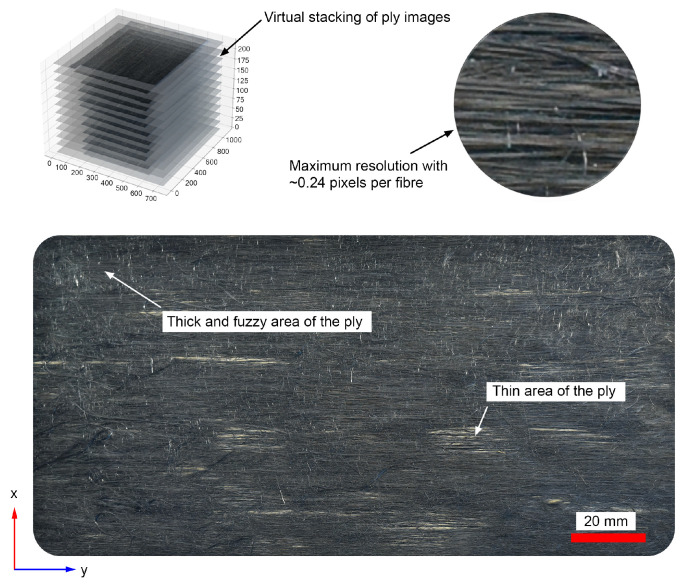
Image acquisition of LS plate ply section taken with Nikon D810 digital single-lens reflex (DSLR) camera [12].

**Figure 4:**
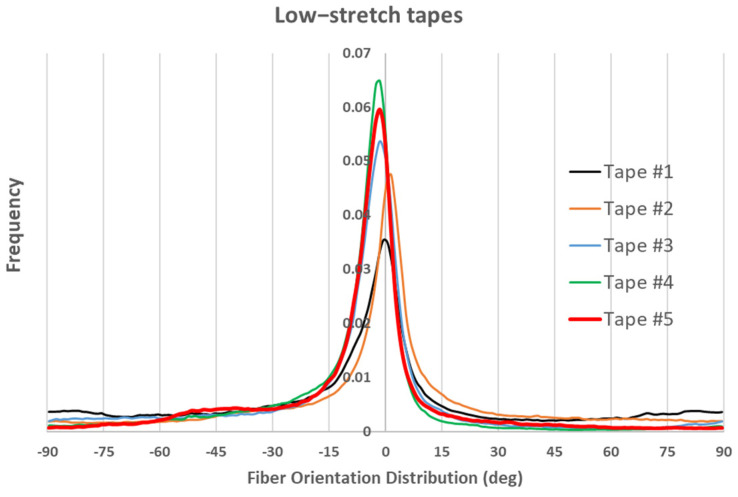
Fiber orientation distribution in five low-stretch tapes.

**Figure 5:**
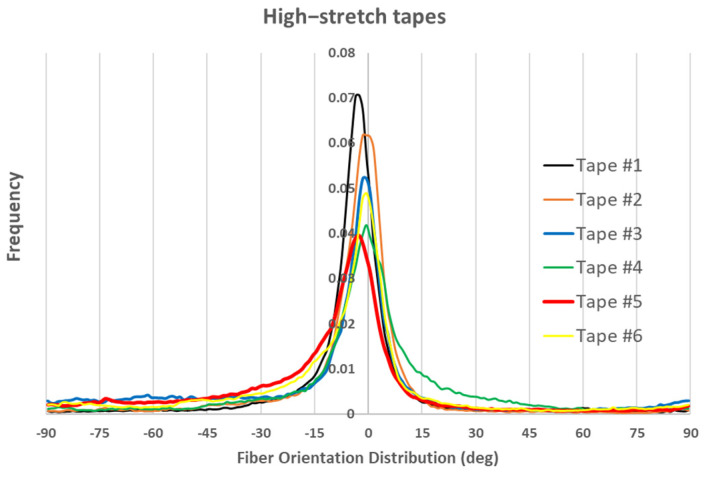
Fiber orientation distribution in six high-stretch tapes.

In addition, optical imaging during plate manufacturing was utilized to analyze FODs within the composite plies (see Figure 3). Images were captured using a calibrated Digital Single-Lens Reflex (DSLR) camera equipped with a polarizing filter to reduce reflection artifacts. A Python-based (Version 3.8.12) automated script processed the images, segmenting them into blocks for orientation analysis using the structure tensor method. The approach analyzes fiber orientation at each pixel by computing the structure tensor, extracting its dominant eigenvector to determine the local orientation. This approach efficiently extracted alignment metrics and visualized FODs in binned plots. The detailed explanation of this analysis can be found in Ref. [12], however, the resulting fiber orientation distribution for some of the low-stretch and high-stretch tapes that are used for manufacturing the samples can be seen in Figure 4 and Figure 5, respectively.

In the above figures, frequency represents how often fibers are found at a particular orientation within the sample. It indicates the proportion or count of fibers aligned in a specific direction. A clear observation in both figures is that most fibers are oriented within ±10 degrees of the axial direction, with better fiber alignment observed in high-stretch tapes (indicated by a higher frequency within ±10 degrees). Another notable observation is that, for almost all tapes, the maximum frequency does not occur at 0 degrees. This is likely also the case for virgin carbon fiber composite tapes, as perfect alignment is unattainable. However, the most important observation in Figure 4 and Figure 5 is that the fiber orientation distribution is not symmetric with respect to 0 degrees, nor with respect to the angle corresponding to the maximum frequency. To emphasize this point, the fiber orientations for Tape 5 in both figures are plotted with thicker lines, which clearly illustrate the asymmetry. As will be explained later, this lack of symmetry in fiber orientation distribution will contribute to the formation of an unbalanced laminate showing in-plane shear–tensile coupling deformation. Additionally, it should also be noted that as mentioned in the previous section, 12 plies are used to make the laminate (see Figure 3). Each ply has a special distribution of under-aligned and over-aligned fiber areas. As these tapes present different fiber orientation distributions (even if slightly), fiber volume fractions, and thicknesses, the resulting laminate might be non-symmetric with respect to its mid-plane (depending on the arrangement of tapes) showing a bending deformation even under a uniaxial loading.

## 3. Thermo-Mechanical Analysis (TMA)

The coefficients of thermal expansion (CTEs) of the composite are sensitive parameters that significantly impact the level of thermal residual stress when subject to thermal environments. The type of temperature dependency, whether linear or non-linear, plays a crucial role in material behavior and requires detailed investigation. Thermo-Mechanical analysis (TMA) is a technique used to measure CTEs by monitoring dimensional changes as a function of temperature. A sample is subjected to a controlled temperature program while a probe measures its dimensional changes with high precision. This analysis is very cumbersome for carbon fiber composites because carbon fibers exhibit very small expansion coefficients leading to small deformation under thermal loading, which then requires an accurate and careful way to measure the samples dimensions.

In this study, two methods were used to measure the CTEs using a TMA TA Q400 from TA Instruments (New Castle, DE, USA). To measure the axial CTE in the fiber direction and the transverse CTE, perpendicular to the fiber direction, a tensile setup was used with sample dimensions of 20 mm × 3 mm. In the through-thickness direction, for the out-of-plane CTE, a standard compression setup was used with the dimensions of 7 mm × 7 mm. All samples had a thickness dimension of 1.6 mm. Thus, the samples manufactured using different tapes were cut into the required dimensions. The sample was placed in a compression and tensile setup, ensuring consistent contact and accurate measurements during the thermal expansion process. The sample was subjected to controlled heating with a temperature ramp of 3 °C/min, starting from 0 °C and increasing to 100 °C. Measurements were performed in three orientations to comprehensively assess directional thermal expansion:

- Fiber Direction: parallel to the predominant alignment of the fibers.
- Transverse Direction: perpendicular to the fiber alignment within the composite plane.
- Out-of-Plane Direction: normal to the plane of the composite.

In addition to measuring the CTEs for the samples made from carbon staple fiber composites, the in-plane axial CTE of a sample made of virgin UD carbon composites was also experimentally measured.

## 4. Modeling Approach

For UD continuous fiber composite plies, the coefficients of thermal expansion can be calculated using various micromechanical homogenization methods based on fiber and matrix properties, assuming transversely isotropic behavior for the homogenized ply. The prediction of CTEs for carbon staple fiber composites requires further considerations.

As discussed in previous sections, using accurate fiber orientation distribution analysis, carbon staple fiber tapes—despite the use of state-of-the-art alignment techniques—exhibit fiber orientation distributions with particular features. Notably, the fiber orientation distribution does not have its maximum value at 0 degrees, meaning that the majority of fibers are oriented at an angle to the axial direction. Furthermore, fiber orientations are not symmetric with respect to 0 degrees or their maximum value. This asymmetry implies that under a unidirectional axial load, coupled in-plane and shear deformation can be expected.

When stacking these tapes into a laminate, the resulting laminate will exhibit behavior distinct from that of a typical UD laminate. In addition to potential coupling deformations, variations in fiber volume fraction and tape thickness through the laminate’s thickness significantly influence the characterization of carbon staple fiber composites. Fiber orientation distribution differs in each layer, meaning that varying fiber distributions must be considered through the thickness of a laminate made of staple fiber composites. Moreover, due to the non-uniform distribution of fibers, the presence of porosity, and resin-rich areas, the local fiber volume fraction changes across the laminate thickness, likely impacting its bending behavior due to unsymmetric configuration with respect to the mid-plane.

The typical approach to modeling the axial Young’s modulus of carbon staple fiber composites involves a straightforward extension of the rule of mixtures [12,22] as follows:

(1) $$E_{A}=\eta_{0}\eta_{1}V_{f}E_{A}^{f}+V_{m}E_{A}^{m},$$

where $E_{A}$  is axial Young’s modulus of the homogenized composite, $\eta_{0}$  is a correction factor for the fiber orientation, $\eta_{1}$  is a correction factor to take into account the short length of the fibers, typically obtained based on the Cox model [30], $V_{f}$  and $E_{A}^{f}$  are the fiber volume fraction and axial Young’s modulus, while $V_{m}$  and $E_{m}^{f}$  are the corresponding properties for the matrix. $\eta_{0}$  is defined by

(2) $$\eta_{0}=\sum \beta_{i}\cos^{4}\theta_{i},$$

where $\beta_{i}$  is the frequency of the related fiber orientation at angle $\theta_{i}$  (see Figure 4 and Figure 5). Consequently, a perfect fiber orientation distribution will result in a correction factor value of 1. In the current work, the carbon fiber lengths range from 40 mm to 60 mm, and with an average diameter of 7 µm, they have an aspect ratio L/R > 1000. As shown in [29], for such large aspect ratios, the effects of fiber length on the thermo-elastic properties of composites can be neglected, even at low fiber volume fractions. There has been little to no effort to predict other properties of carbon staple fiber composites including coefficients of thermal expansion.

An obvious issue with using Equation (1) to predict the in-plane axial Young’s modulus of carbon staple fiber composites is that it does not account for the transversely isotropic nature of carbon fibers. This omission is significant because the Young’s modulus of carbon fibers is much lower in the transverse direction, and most fibers in staple fiber composites are oriented at angles other than the axial direction. Furthermore, the equation relies on an averaging procedure for fiber orientations and volume fractions. For example, when the frequencies of fiber orientations at angles $\theta_{i}$  and $-\theta_{i}$  are different, Equation (2) yields the same value for the correction factor as when the frequencies of fiber orientations at angles $\theta_{i}$  and $-\theta_{i}$  are identical, assuming with the same sum value, neglecting the potential coupling between in-plane axial and shear deformations. Additionally, the inability to account for tape thickness, fiber volume fraction, and orientation disregards critical microscopic characteristics of carbon staple fiber composites that could significantly influence their thermo-mechanical response.

Here, to predict all thermo-elastic properties while considering microstructural information, a methodology based on a laminate analogy will be adopted. First, for each bundle of fibers embedded in the matrix and oriented at a specific angle (grain), the following formula will be used to determine the unidirectional transversely isotropic properties in that orientation [31]:

(3) $$E_{A}=V_{f}E_{A}^{f}+V_{m}E_{A}^{m}+2\lambda {\left(v_{A}^{m}-v_{A}^{f}\right)}^{2}V_{f}V_{m},$$

(4) $$v_{A}=V_{f}v_{A}^{f}+V_{m}v_{A}^{m}-\frac{\lambda }{2}(v_{A}^{f}-v_{A}^{m})\left(\frac{1}{k_{T}^{f}}-\frac{1}{k_{T}^{m}}\right)V_{f}V_{m},$$

(5) $$\frac{1}{k_{T}}\equiv \frac{2\left(1-v_{T}\right)}{E_{T}}-\frac{4{\left(v_{A}\right)}^{2}}{E_{A}}=\frac{1}{k_{T}^{*}}-\frac{\lambda }{2}{\left(\frac{1}{k_{T}^{f}}-\frac{1}{k_{T}^{m}}\right)}^{2}V_{f}V_{m},$$

where

(6) $$\begin{array}{l} \frac{1}{k_{T}^{m}}=\frac{2\left(1-v_{T}^{m}\right)}{E_{T}^{m}}-\frac{4{\left(v_{A}^{m}\right)}^{2}}{E_{A}^{m}},\;\frac{1}{k_{T}^{f}}=\frac{2\left(1-v_{T}^{f}\right)}{E_{T}^{f}}-\frac{4{\left(v_{A}^{f}\right)}^{2}}{E_{A}^{f}},\\ \frac{1}{\lambda }=\frac{1}{2}\left(\frac{1}{\mu_{T}^{m}}+\frac{V_{f}}{k_{T}^{m}}+\frac{V_{m}}{k_{T}^{f}}\right),\;\frac{1}{k_{T}^{*}}=\frac{V_{f}}{k_{T}^{f}}+\frac{V_{m}}{k_{T}^{m}}, \end{array},$$

and where E  denotes Young’s modulus, μ  denotes shear modulus, v  denotes Poisson’s ratio, and *k* denotes transverse bulk modulus. The superscripts *f* and *m* denote fiber and matrix properties, respectively, while the subscripts *A* and *T* denote in-plane axial and transverse directions, respectively. The above formulations are superior to the simple rule of mixtures, as they are derived based on concentric cylindrical models, which account for stress transfer between the fiber and matrix more accurately, along with the potential effects of fiber and matrix transverse isotropy.

The effective shear modulus will then be written as follows:

(7) $$\begin{array}{l} \mu_{T}=V_{f}\mu_{T}^{f}+V_{m}\mu_{T}^{m}-\lambda_{I}{\left(\mu_{T}^{f}-\mu_{T}^{m}\right)}^{2}V_{f}V_{m},\\ \mu_{A}=V_{f}\mu_{A}^{f}+V_{m}\mu_{A}^{m}-\lambda_{II}{\left(\mu_{A}^{f}-\mu_{A}^{m}\right)}^{2}V_{f}V_{m}, \end{array},$$

where

(8) $$\begin{array}{l} \frac{1}{\lambda_{I}}=V_{m}\mu_{T}^{f}+V_{f}\mu_{T}^{m}+\frac{k_{T}^{m}\mu_{T}^{m}}{k_{T}^{m}+2\mu_{T}^{m}},\\ \frac{1}{\lambda_{II}}=\mu_{A}^{m}\left(1+V_{f}\right)+V_{m}\mu_{A}^{f}. \end{array}$$

The axial $\alpha_{A}$  and transverse $\alpha_{T}$  coefficients of thermal expansion can be defined as follows:

(9) $$\alpha_{A}=\frac{1}{E_{A}}\left[\begin{array}{l} V_{f}E_{A}^{f}\alpha_{A}^{f}+V_{m}E_{A}^{m}\alpha_{A}^{m}\\ +2\lambda \left(v_{A}^{m}-v_{A}^{f}\right)V_{f}V_{m}\left(\alpha_{T}^{m}+v_{A}^{m}\alpha_{A}^{m}-\alpha_{T}^{f}-v_{A}^{f}\alpha_{A}^{f}\right) \end{array}\right],$$

(10) $$\begin{array}{l} \alpha_{T}=-\nu_{A}\alpha_{A}+\left(\alpha_{T}^{f}+v_{A}^{f}\alpha_{A}^{f}\right)V_{f}+\left(\alpha_{T}^{m}+v_{A}^{m}\alpha_{A}^{m}\right)V_{m}\\ +\frac{\lambda }{2}\left(\frac{1}{k_{T}^{f}}-\frac{1}{k_{T}^{m}}\right)V_{f}V_{m}\left(\alpha_{T}^{m}+v_{A}^{m}\alpha_{A}^{m}-\alpha_{T}^{f}-v_{A}^{f}\alpha_{A}^{f}\right). \end{array}$$

Finally, by considering that the homogenized system is transversely isotropic, the in-plane transverse Young’s modulus $E_{T}$  and Poisson’s ratio $v_{T}$  can be obtained when having $\mu_{T}$  and $k_{T}$ , as well as the following relation:

(11) $$E_{T}=2\mu_{T}\left(1+v_{T}\right).$$

With the homogenized effective properties for each bundle of fibers in the matrix (grain) oriented at a specific angle within a tape, it becomes possible to approximate the homogenized properties of the tape based on its fiber orientation distribution as follows:

(12) $$[\bar{C}]=\sum \beta_{i}{\left({\left[R^{\left(i\right)}\right]}^{T}[s^{\left(i\right)}][R^{\left(i\right)}]\right)}^{-1},[\bar{S}]={\left[\bar{C}\right]}^{-1},$$

(13) $$\left\{\bar{\alpha }\right\}=[\bar{S}]\left(\sum \beta_{i}{\left[R^{\left(i\right)}\right]}^{-1}{\left[s^{\left(i\right)}\right]}^{-1}\left\{\alpha^{\left(i\right)}\right\}\right),$$

where $\left[\bar{S}\right]$  and $\left\{\bar{\alpha }\right\}$  are the *tape* homogenized compliance matrix and thermal expansion vector, respectively, and $\beta_{i}$  is the frequency of the related bundle of fibers in the matrix at angle $\theta_{i}$  in the tape. The $\left[s^{\left(i\right)}\right]$  and $\left\{\alpha^{\left(i\right)}\right\}$  are, respectively, the homogenized compliance matrix and CTE vector of the grain oriented at angle $\theta_{i}$  in their local coordinate system, and $\left[R^{\left(i\right)}\right]$  is the transformation matrix. These terms can be defined as follows, in terms of effective homogenized properties of each grain (*i*):

(14) $$[s^{\left(i\right)}]=\left[\begin{array}{cccccc} \frac{1}{E_{A}^{\left(i\right)}} & -\frac{v_{A}^{\left(i\right)}}{E_{A}^{\left(i\right)}} & -\frac{v_{A}^{\left(i\right)}}{E_{A}^{\left(i\right)}} & 0 & 0 & 0\\ -\frac{v_{A}^{\left(i\right)}}{E_{A}^{\left(i\right)}} & \frac{1}{E_{T}^{\left(i\right)}} & -\frac{v_{A}^{\left(i\right)}}{E_{A}^{\left(i\right)}} & 0 & 0 & 0\\ -\frac{v_{A}^{\left(i\right)}}{E_{A}^{\left(i\right)}} & -\frac{v_{A}^{\left(i\right)}}{E_{A}^{\left(i\right)}} & \frac{1}{E_{T}^{\left(i\right)}} & 0 & 0 & 0\\ 0 & 0 & 0 & \frac{1}{\mu_{T}^{\left(i\right)}} & 0 & 0\\ 0 & 0 & 0 & 0 & \frac{1}{\mu_{A}^{\left(i\right)}} & 0\\ 0 & 0 & 0 & 0 & 0 & \frac{1}{\mu_{A}^{\left(i\right)}} \end{array}\right],\left\{\alpha^{\left(i\right)}\right\}=\left\{\begin{array}{c} \alpha_{A}^{\left(i\right)}\\ \alpha_{T}^{\left(i\right)}\\ \alpha_{T}^{\left(i\right)}\\ 0\\ 0\\ 0 \end{array}\right\},$$

(15) $$[R^{\left(i\right)}]=\left[\begin{array}{cccccc} m^{2} & n^{2} & 0 & 0 & 0 & 2mn\\ n^{2} & m^{2} & 0 & 0 & 0 & -2mn\\ 0 & 0 & 1 & 0 & 0 & 0\\ 0 & 0 & 0 & m & -n & 0\\ 0 & 0 & 0 & n & m & 0\\ -mn & mn & 0 & 0 & 0 & m^{2}-n^{2} \end{array}\right],\;m=\cos(\theta_{i}),n=\sin(\theta_{i}).$$

In Equation (14), it is assumed that each grain oriented at angle $\theta_{i}$  is transversely isotropic. It is clear that if we assume the same material properties and volume fraction for each grain, the homogenized compliance matrix $\left[s^{\left(i\right)}\right]$  and CTE vector $\left\{\alpha^{\left(i\right)}\right\}$  will be the same for all grains. The difference arises solely from variations in fiber orientation $\left[R^{\left(i\right)}\right]$  and the frequency of that orientation $\beta_{i}$ . One can consider a statistical distribution for fiber volume fractions, fiber/matrix material properties, and fiber length, leading to different values for the homogenized compliance matrix $\left[s^{\left(i\right)}\right]$  and CTE vector $\left\{\alpha^{\left(i\right)}\right\}$  for each grain, in order to account for potential variations in void distribution, fiber length, and material properties. It is clear that due to a non-symmetric distribution of fibers with respect to the zero-degree in the fiber orientation tensor, the tape will not be balanced unlike a typical continuous fiber UD tape.

Another feature of carbon staple fiber composites is that each tape has a different fiber orientation distribution and possibly varying thickness. Therefore, when stacking them to achieve the desired laminate thickness, the variation in fiber orientation throughout the thickness must also be considered. In order to calculate the thermo-elastic response of staple fiber composite samples at the macroscopic level, the enhanced classical laminate theory (ECLPT) is adopted [32,33]. The key aspect regarding the use of the enhanced laminate theory is the possibility of considering three-dimensional solutions for evaluating the triaxial response of composite materials where uniform normal stresses and/or shear tractions are considered for the upper and lower surfaces. This solution is applicable for both symmetric and non-symmetric multi-layered composites with *N* plies where each ply may have different mechanical properties. The interface between the adjacent layers is considered to be perfectly bonded and the continuity of both stress and displacement fields are satisfied at the interface between the plies. In this work, we assume that carbon staple fiber composites form a symmetric laminate, although it is possible to consider non-symmetric lay-ups as well. Using the ECLPT, for a symmetric laminate made of staple fiber composite tapes, subject to the uniform effective triaxial $\left({\tilde{\sigma }}_{A},{\tilde{\sigma }}_{T},{\tilde{\sigma }}_{t}\right)$  and in-plane and out-of-plane shear loads $\left({\tilde{\tau }}_{A},{\tilde{\tau }}_{t},{\tilde{\tau }}_{a}\right)$ , the out-of-plane shear and normal stresses $\left({\tilde{\sigma }}_{t},{\tilde{\tau }}_{t},{\tilde{\tau }}_{a}\right)$  as well as the in-plane strains $\left({\tilde{\varepsilon }}_{A},{\tilde{\varepsilon }}_{T},{\tilde{\gamma }}_{A}\right)$  have the same values for all tapes in the laminate. Therefore, the following strain–stress equations can be applied to each tape (*i*) of the laminate in the global coordinate system:

(16) $$\left\{\begin{array}{c} {\tilde{\varepsilon }}_{A}\\ {\tilde{\varepsilon }}_{T}\\ {\bar{\varepsilon }}_{t}^{\left(i\right)}\\ {\bar{\gamma }}_{t}^{\left(i\right)}\\ {\bar{\gamma }}_{a}^{\left(i\right)}\\ {\tilde{\gamma }}_{A} \end{array}\right\}=\underset{\left[{\bar{S}}^{\left(i\right)}\right]}{\underset{︸}{\left[\begin{array}{cccccc} {\bar{S}}_{11}^{\left(i\right)} & {\bar{S}}_{12}^{\left(i\right)} & {\bar{S}}_{13}^{\left(i\right)} & 0 & 0 & {\bar{S}}_{16}^{\left(i\right)}\\ {\bar{S}}_{12}^{\left(i\right)} & {\bar{S}}_{22}^{\left(i\right)} & {\bar{S}}_{23}^{\left(i\right)} & 0 & 0 & {\bar{S}}_{26}^{\left(i\right)}\\ {\bar{S}}_{13}^{\left(i\right)} & {\bar{S}}_{23}^{\left(i\right)} & {\bar{S}}_{33}^{\left(i\right)} & 0 & 0 & {\bar{S}}_{36}^{\left(i\right)}\\ 0 & 0 & 0 & {\bar{S}}_{44}^{\left(i\right)} & {\bar{S}}_{45}^{\left(i\right)} & 0\\ 0 & 0 & 0 & {\bar{S}}_{45}^{\left(i\right)} & {\bar{S}}_{55}^{\left(i\right)} & 0\\ {\bar{S}}_{16}^{\left(i\right)} & {\bar{S}}_{26}^{\left(i\right)} & {\bar{S}}_{36}^{\left(i\right)} & 0 & 0 & {\bar{S}}_{66}^{\left(i\right)} \end{array}\right]}}\left\{\begin{array}{c} {\bar{\sigma }}_{A}^{\left(i\right)}\\ {\bar{\sigma }}_{T}^{\left(i\right)}\\ {\tilde{\sigma }}_{t}\\ {\tilde{\tau }}_{t}\\ {\tilde{\tau }}_{a}\\ {\bar{\tau }}_{A}^{\left(i\right)} \end{array}\right\}+\underset{\left\{{\bar{\alpha }}^{\left(i\right)}\right\}}{\underset{︸}{\left\{\begin{array}{c} {\bar{\alpha }}_{1}^{\left(i\right)}\\ {\bar{\alpha }}_{2}^{\left(i\right)}\\ {\bar{\alpha }}_{3}^{\left(i\right)}\\ 0\\ 0\\ {\bar{\alpha }}_{6}^{\left(i\right)} \end{array}\right\}}}\Delta T,$$

where the overbar on [*S*] and other parameters indicates that they represent tape properties (defined by Equations (12) and (13)), while the tilde corresponds to laminate properties and their associated values. Note that the uniform temperature difference is defined by $\Delta T=T-T_{0}$  where T  is the current temperature and $T_{0}$  is the reference temperature for which the strains and stresses are zero with no internal or imposed external stresses.

The inverted form of (16) can be written as follows in terms of tape stiffness parameters:

(17) $$\left\{\begin{array}{c} {\bar{\sigma }}_{A}^{\left(i\right)}\\ {\bar{\sigma }}_{T}^{\left(i\right)}\\ {\tilde{\sigma }}_{t}\\ {\tilde{\tau }}_{t}\\ {\tilde{\tau }}_{a}\\ {\bar{\tau }}_{A}^{\left(i\right)} \end{array}\right\}=\underset{\left[{\bar{C}}^{\left(i\right)}\right]}{\underset{︸}{\left[\begin{array}{cccccc} {\bar{C}}_{11}^{\left(i\right)} & {\bar{C}}_{12}^{\left(i\right)} & {\bar{C}}_{13}^{\left(i\right)} & 0 & 0 & {\bar{C}}_{16}^{\left(i\right)}\\ {\bar{C}}_{12}^{\left(i\right)} & {\bar{C}}_{22}^{\left(i\right)} & {\bar{C}}_{23}^{\left(i\right)} & 0 & 0 & {\bar{C}}_{26}^{\left(i\right)}\\ {\bar{C}}_{13}^{\left(i\right)} & {\bar{C}}_{23}^{\left(i\right)} & {\bar{C}}_{33}^{\left(i\right)} & 0 & 0 & {\bar{C}}_{36}^{\left(i\right)}\\ 0 & 0 & 0 & {\bar{C}}_{44}^{\left(i\right)} & {\bar{C}}_{45}^{\left(i\right)} & 0\\ 0 & 0 & 0 & {\bar{C}}_{45}^{\left(i\right)} & {\bar{C}}_{55}^{\left(i\right)} & 0\\ {\bar{C}}_{16}^{\left(i\right)} & {\bar{C}}_{26}^{\left(i\right)} & {\bar{C}}_{36}^{\left(i\right)} & 0 & 0 & {\bar{C}}_{66}^{\left(i\right)} \end{array}\right]}}\left\{\begin{array}{c} {\tilde{\varepsilon }}_{A}\\ {\tilde{\varepsilon }}_{T}\\ {\bar{\varepsilon }}_{t}^{\left(i\right)}\\ {\bar{\gamma }}_{t}^{\left(i\right)}\\ {\bar{\gamma }}_{a}^{\left(i\right)}\\ {\tilde{\gamma }}_{A} \end{array}\right\}-\underset{\left\{{\bar{U}}^{\left(i\right)}\right\}}{\underset{︸}{\left\{\begin{array}{c} {\bar{U}}_{1}^{\left(i\right)}\\ {\bar{U}}_{2}^{\left(i\right)}\\ {\bar{U}}_{3}^{\left(i\right)}\\ 0\\ 0\\ {\bar{U}}_{6}^{\left(i\right)} \end{array}\right\}}}\Delta T,$$

where

(18) $$[{\bar{C}}^{\left(i\right)}]={\left[{\bar{S}}^{\left(i\right)}\right]}^{-1},\left\{{\bar{U}}^{\left(i\right)}\right\}={\left[{\bar{S}}^{\left(i\right)}\right]}^{-1}\left\{{\bar{\alpha }}^{\left(i\right)}\right\}.$$

The Equation (17) can be rearranged to have uniform strain/stress values (for the laminate) on the right-hand side, as follows:

(19) $$\begin{array}{l} {\bar{\sigma }}_{A}^{\left(i\right)}={\bar{\Omega }}_{11}^{\left(i\right)}{\tilde{\varepsilon }}_{A}+{\bar{\Omega }}_{12}^{\left(i\right)}{\tilde{\varepsilon }}_{T}+{\bar{\Omega }}_{13}^{\left(i\right)}{\tilde{\sigma }}_{t}+{\bar{\Omega }}_{16}^{\left(i\right)}{\tilde{\gamma }}_{A}-{\bar{W}}_{1}^{\left(i\right)}\Delta T,\\ {\bar{\sigma }}_{T}^{\left(i\right)}={\bar{\Omega }}_{12}^{\left(i\right)}{\tilde{\varepsilon }}_{A}+{\bar{\Omega }}_{22}^{\left(i\right)}{\tilde{\varepsilon }}_{T}+{\bar{\Omega }}_{23}^{\left(i\right)}{\tilde{\sigma }}_{t}+{\bar{\Omega }}_{26}^{\left(i\right)}{\tilde{\gamma }}_{A}-{\bar{W}}_{2}^{\left(i\right)}\Delta T,\\ {\bar{\varepsilon }}_{t}^{\left(i\right)}=-{\bar{\Omega }}_{13}^{\left(i\right)}{\tilde{\varepsilon }}_{A}-{\bar{\Omega }}_{23}^{\left(i\right)}{\tilde{\varepsilon }}_{T}+{\bar{\Omega }}_{33}^{\left(i\right)}{\tilde{\sigma }}_{t}-{\bar{\Omega }}_{36}^{\left(i\right)}{\tilde{\gamma }}_{A}+{\bar{W}}_{3}^{\left(i\right)}\Delta T,\\ {\bar{\gamma }}_{t}^{\left(i\right)}={\bar{\Omega }}_{44}^{\left(i\right)}{\tilde{\tau }}_{t}+{\bar{\Omega }}_{45}^{\left(i\right)}{\tilde{\tau }}_{a},\\ {\bar{\gamma }}_{a}^{\left(i\right)}={\bar{\Omega }}_{45}^{\left(i\right)}{\tilde{\tau }}_{t}+{\bar{\Omega }}_{55}^{\left(i\right)}{\tilde{\tau }}_{a},\\ {\bar{\tau }}_{A}^{\left(i\right)}={\bar{\Omega }}_{16}^{\left(i\right)}{\tilde{\varepsilon }}_{A}+{\bar{\Omega }}_{26}^{\left(i\right)}{\tilde{\varepsilon }}_{T}+{\bar{\Omega }}_{36}^{\left(i\right)}{\tilde{\sigma }}_{t}+{\bar{\Omega }}_{66}^{\left(i\right)}{\tilde{\gamma }}_{A}-{\bar{W}}_{6}^{\left(i\right)}\Delta T, \end{array},$$

where

(20) $$\begin{array}{l} {\bar{\Omega }}_{11}^{\left(i\right)}={\bar{C}}_{11}^{\left(i\right)}-\frac{{\bar{C}}_{13}^{\left(i\right)}{\bar{C}}_{13}^{\left(i\right)}}{{\bar{C}}_{33}^{\left(i\right)}},\;{\bar{\Omega }}_{12}^{\left(i\right)}={\bar{C}}_{12}^{\left(i\right)}-\frac{{\bar{C}}_{13}^{\left(i\right)}{\bar{C}}_{23}^{\left(i\right)}}{{\bar{C}}_{33}^{\left(i\right)}},\\ {\bar{\Omega }}_{13}^{\left(i\right)}=\frac{{\bar{C}}_{13}^{\left(i\right)}}{{\bar{C}}_{33}^{\left(i\right)}},\;{\bar{\Omega }}_{16}^{\left(i\right)}={\bar{C}}_{16}^{\left(i\right)}-\frac{{\bar{C}}_{13}^{\left(i\right)}{\bar{C}}_{36}^{\left(i\right)}}{{\bar{C}}_{33}^{\left(i\right)}},\\ {\bar{\Omega }}_{22}^{\left(i\right)}={\bar{C}}_{22}^{\left(i\right)}-\frac{{\bar{C}}_{23}^{\left(i\right)}{\bar{C}}_{23}^{\left(i\right)}}{{\bar{C}}_{33}^{\left(i\right)}},\;{\bar{\Omega }}_{23}^{\left(i\right)}=\frac{{\bar{C}}_{23}^{\left(i\right)}}{{\bar{C}}_{33}^{\left(i\right)}},\\ {\bar{\Omega }}_{26}^{\left(i\right)}={\bar{C}}_{26}^{\left(i\right)}-\frac{{\bar{C}}_{23}^{\left(i\right)}{\bar{C}}_{36}^{\left(i\right)}}{{\bar{C}}_{33}^{\left(i\right)}},\;{\bar{\Omega }}_{33}^{\left(i\right)}=\frac{1}{{\bar{C}}_{33}^{\left(i\right)}},\;{\bar{\Omega }}_{36}^{\left(i\right)}=\frac{{\bar{C}}_{36}^{\left(i\right)}}{{\bar{C}}_{33}^{\left(i\right)}},\\ {\bar{\Omega }}_{66}^{\left(i\right)}={\bar{C}}_{66}^{\left(i\right)}-\frac{{\bar{C}}_{36}^{\left(i\right)}{\bar{C}}_{36}^{\left(i\right)}}{{\bar{C}}_{33}^{\left(i\right)}},\;{\bar{\Omega }}_{44}^{\left(i\right)}=\frac{{\bar{C}}_{55}^{\left(i\right)}}{{\bar{C}}_{44}^{\left(i\right)}{\bar{C}}_{55}^{\left(i\right)}-{\bar{C}}_{45}^{\left(i\right)}{\bar{C}}_{45}^{\left(i\right)}},\\ {\bar{\Omega }}_{45}^{\left(i\right)}=-\frac{{\bar{C}}_{45}^{\left(i\right)}}{{\bar{C}}_{44}^{\left(i\right)}{\bar{C}}_{55}^{\left(i\right)}-{\bar{C}}_{45}^{\left(i\right)}{\bar{C}}_{45}^{\left(i\right)}},\;{\bar{\Omega }}_{55}^{\left(i\right)}=\frac{{\bar{C}}_{44}^{\left(i\right)}}{{\bar{C}}_{44}^{\left(i\right)}{\bar{C}}_{55}^{\left(i\right)}-{\bar{C}}_{45}^{\left(i\right)}{\bar{C}}_{45}^{\left(i\right)}},\\ {\bar{W}}_{1}^{\left(i\right)}={\bar{U}}_{1}^{\left(i\right)}-\frac{{\bar{C}}_{13}^{\left(i\right)}{\bar{U}}_{3}^{\left(i\right)}}{{\bar{C}}_{33}^{\left(i\right)}},\;{\bar{W}}_{2}^{\left(i\right)}={\bar{U}}_{2}^{\left(i\right)}-\frac{{\bar{C}}_{23}^{\left(i\right)}{\bar{U}}_{3}^{\left(i\right)}}{{\bar{C}}_{33}^{\left(i\right)}},\\ {\bar{W}}_{3}^{\left(i\right)}=\frac{{\bar{U}}_{3}^{\left(i\right)}}{{\bar{C}}_{33}^{\left(i\right)}},\;{\bar{W}}_{6}^{\left(i\right)}={\bar{U}}_{6}^{\left(i\right)}-\frac{{\bar{C}}_{36}^{\left(i\right)}{\bar{U}}_{3}^{\left(i\right)}}{{\bar{C}}_{33}^{\left(i\right)}}. \end{array}$$

The corresponding effective in-plane stresses and the out-of-plane normal and shear strains for the laminate can be defined by the following averages:

(21) $$\begin{array}{l} {\tilde{\sigma }}_{A}=\frac{1}{h}\sum_{i=1}^{N}h_{i}{\bar{\sigma }}_{A}^{\left(i\right)},\;{\tilde{\sigma }}_{T}=\frac{1}{h}\sum_{i=1}^{N}h_{i}{\bar{\sigma }}_{T}^{\left(i\right)},\;{\tilde{\tau }}_{A}=\frac{1}{h}\sum_{i=1}^{N}h_{i}{\bar{\tau }}_{A}^{\left(i\right)},\\ {\tilde{\varepsilon }}_{t}=\frac{1}{h}\sum_{i=1}^{N}h_{i}{\bar{\varepsilon }}_{t}^{\left(i\right)},\;{\tilde{\gamma }}_{t}=\frac{1}{h}\sum_{i=1}^{N}h_{i}{\bar{\gamma }}_{t}^{\left(i\right)},\;{\tilde{\gamma }}_{a}=\frac{1}{h}\sum_{i=1}^{N}h_{i}{\bar{\gamma }}_{a}^{\left(i\right)}, \end{array}$$

where *h* is the laminate thickness, $h_{i}$  represents the thickness of the tape (*i*), and *N* is the number of tapes considered in the laminate. The formulation then demonstrates how the effects of tapes with different thicknesses can be incorporated when predicting the properties of staple fiber composite laminates.

Applying the Equation (21) to the Equation (19), the following effective stress–strain–temperature relations can be obtained:

(22) $$\begin{array}{l} {\tilde{\sigma }}_{A}={\tilde{\Omega }}_{11}{\tilde{\varepsilon }}_{A}+{\tilde{\Omega }}_{12}\varepsilon_{T}+{\tilde{\Omega }}_{13}\sigma_{t}+{\tilde{\Omega }}_{16}\gamma_{A}-{\tilde{W}}_{1}\Delta T,\\ {\tilde{\sigma }}_{T}={\tilde{\Omega }}_{12}{\tilde{\varepsilon }}_{A}+{\tilde{\Omega }}_{22}\varepsilon_{T}+{\tilde{\Omega }}_{23}\sigma_{t}+{\tilde{\Omega }}_{26}\gamma_{A}-{\tilde{W}}_{2}\Delta T,\\ {\tilde{\varepsilon }}_{t}=-{\tilde{\Omega }}_{13}{\tilde{\varepsilon }}_{A}-{\tilde{\Omega }}_{23}\varepsilon_{T}+{\tilde{\Omega }}_{33}\sigma_{t}-{\tilde{\Omega }}_{36}\gamma_{A}+{\tilde{W}}_{3}\Delta T,\\ {\tilde{\gamma }}_{t}={\tilde{\Omega }}_{44}{\tilde{\tau }}_{t}+{\tilde{\Omega }}_{45}{\tilde{\tau }}_{a},\\ {\tilde{\gamma }}_{a}={\tilde{\Omega }}_{45}{\tilde{\tau }}_{t}+{\tilde{\Omega }}_{55}{\tilde{\tau }}_{a},\\ {\tilde{\tau }}_{A}={\tilde{\Omega }}_{16}{\tilde{\varepsilon }}_{A}+{\tilde{\Omega }}_{26}{\tilde{\varepsilon }}_{T}+{\tilde{\Omega }}_{36}{\tilde{\sigma }}_{t}+{\tilde{\Omega }}_{66}{\tilde{\gamma }}_{A}-{\tilde{W}}_{6}\Delta T, \end{array}$$

where

(23) $${\tilde{\Omega }}_{mn}=\frac{1}{h}\sum_{i=1}^{N}h_{i}{\bar{\Omega }}_{mn}^{\left(i\right)},\;{\tilde{W}}_{m}=\frac{1}{h}\sum_{i=1}^{N}h_{i}{\bar{W}}_{m}^{\left(i\right)}\;\mathrm{where}\;m,n=1..6\;.$$

The Equation (22) can then be easily rearranged to have all the effective strain terms on the left-hand side. The detail of such operations will not be provided here but the readers can refer to Ref. [34] for details. Finally, the effective stress–strain relations for the carbon staple fiber laminate can be written as follows:

(24) $$\left\{\begin{array}{c} {\tilde{\varepsilon }}_{A}\\ {\tilde{\varepsilon }}_{T}\\ {\tilde{\varepsilon }}_{t}\\ {\tilde{\gamma }}_{t}\\ {\tilde{\gamma }}_{a}\\ {\tilde{\gamma }}_{A} \end{array}\right\}=\underset{\left[\tilde{S}\right]}{\underset{︸}{\left[\begin{array}{cccccc} {\tilde{S}}_{11} & {\tilde{S}}_{12} & {\tilde{S}}_{13} & 0 & 0 & {\tilde{S}}_{16}\\ {\tilde{S}}_{12} & {\tilde{S}}_{22} & {\tilde{S}}_{23} & 0 & 0 & {\tilde{S}}_{26}\\ {\tilde{S}}_{13} & {\tilde{S}}_{23} & {\tilde{S}}_{33} & 0 & 0 & {\tilde{S}}_{36}\\ 0 & 0 & 0 & {\tilde{S}}_{44} & {\tilde{S}}_{45} & 0\\ 0 & 0 & 0 & {\tilde{S}}_{45} & {\tilde{S}}_{55} & 0\\ {\tilde{S}}_{16} & {\tilde{S}}_{26} & {\tilde{S}}_{36} & 0 & 0 & {\tilde{S}}_{66} \end{array}\right]}}\left\{\begin{array}{c} {\tilde{\sigma }}_{A}\\ {\tilde{\sigma }}_{T}\\ {\tilde{\sigma }}_{t}\\ {\tilde{\tau }}_{t}\\ {\tilde{\tau }}_{a}\\ {\tilde{\tau }}_{A} \end{array}\right\}+\underset{\left\{\tilde{\alpha }\right\}}{\underset{︸}{\left\{\begin{array}{c} {\tilde{\alpha }}_{1}\\ {\tilde{\alpha }}_{2}\\ {\tilde{\alpha }}_{3}\\ 0\\ 0\\ {\tilde{\alpha }}_{6} \end{array}\right\}}}\Delta T,$$

where $\left[\tilde{S}\right]$  and $\left\{\tilde{\alpha }\right\}$  are the homogenized laminate compliance matrix and thermal expansion vector, respectively. The well-known laminate thermo-elastic constants can then be defined in terms of the compliance matrix and thermal expansion vector components as follows:

(25) $$\begin{array}{l} {\tilde{E}}_{A}=\frac{1}{{\tilde{S}}_{11}},\;{\tilde{v}}_{A}=-\frac{{\tilde{S}}_{12}}{{\tilde{S}}_{11}},\;{\tilde{v}}_{a}=-\frac{{\tilde{S}}_{13}}{{\tilde{S}}_{11}},\;{\tilde{\lambda }}_{A}=-\frac{{\tilde{S}}_{16}}{{\tilde{S}}_{11}},\;{\tilde{\alpha }}_{A}={\tilde{\alpha }}_{1},\\ {\tilde{E}}_{T}=\frac{1}{{\tilde{S}}_{22}},\;{\tilde{E}}_{t}=\frac{1}{{\tilde{S}}_{33}},\;{\tilde{v}}_{t}=-\frac{{\tilde{S}}_{23}}{{\tilde{S}}_{22}},\;{\tilde{\lambda }}_{T}=-\frac{{\tilde{S}}_{26}}{{\tilde{S}}_{11}},\;{\tilde{\alpha }}_{T}={\tilde{\alpha }}_{2},\\ {\tilde{\lambda }}_{t}=-\frac{{\tilde{S}}_{36}}{{\tilde{S}}_{11}},\;{\tilde{\mu }}_{A}=\frac{1}{{\tilde{S}}_{66}},\;{\tilde{\mu }}_{a}=\frac{1}{{\tilde{S}}_{55}},\;{\tilde{\mu }}_{t}=\frac{1}{{\tilde{S}}_{44}},\;{\tilde{\alpha }}_{t}={\tilde{\alpha }}_{3},\\ {\tilde{\lambda }}_{s}=-\frac{{\tilde{S}}_{45}}{{\tilde{S}}_{55}},\;{\tilde{\alpha }}_{s}={\tilde{\alpha }}_{6}, \end{array}$$

where the tilde sign emphasizes that they are *laminate* effective properties and, the same as before, the parameters *E*, *G*, *υ*, and *α* specify, respectively, Young’s moduli, shear moduli, Poisson’s ratios, and coefficients of thermal expansion. The parameters $\tilde{\lambda }$  are ratios associated with the effect of shear stress on in-plane and transverse shear strains. The upper-case subscripts *A* and *T* are attached to axial and transverse thermo-elastic constants to specify that they refer to axial and transverse deformations while the corresponding lower case subscripts (*a* and *t*) denote thermo-elastic constants that involve through-thickness stress and deformations.

In the current work, a perfect bond between the fibers and matrix is assumed. A more comprehensive failure prediction model would require considering imperfect fiber/matrix interfaces.

## 5. Results and Discussions

The proposed model for predicting the CTEs of carbon staple fiber composites takes the material properties of the fiber and matrix as input parameters. It is evident that these properties might be affected after recycling or during processing. The methodology described above can account for a statistical distribution of fiber and matrix properties, as well as other geometrical parameters. However, in this study, the fiber and matrix thermoelastic properties are assumed to be constant. Table 2 shows the input values for the fiber and matrix materials, directly taken from manufacturer data sheets for the carbon fiber T700S-C-12K-50C and the epoxy resin system EPIKOTE^TM^ 05545/EPIKURE^TM^ 778/EPIKURE^TM^, wherever available. It is noted that the Young’s modulus of 2.76 GPa for the matrix system used in this study may be overestimated due to the effect of toughening agents and adhesion promoters, which could lower glass transition temperature and potentially reduce the Young’s modulus.

### 5.1. Experimental Results

Table 3 presents the experimentally measured thermal expansion coefficients for the virgin laminate, as well as for low-stretched and high-stretched carbon staple fiber composites. The average values of these measurements are highlighted in bold. Notably, for the virgin laminate, only the axial thermal expansion coefficient was measured. The samples have varying volume fractions. Details on the variation of sample dimensions with temperature and the temperature range used for CTE evaluation are provided in the Appendix A figures.

The average results in Table 3 for in-plane transverse and out-of-plane CTEs reveal that the assumption of a transversely isotropic material might not be valid, especially for low-stretched carbon staple fiber composites. The material does not exhibit symmetrical behavior in the T and t directions, indicating orthotropic behavior. Furthermore, the CTE in the axial fiber direction is ten times lower than the fiber’s initial value. This is unexpected, as the composite was anticipated to have a higher axial CTE than the fiber itself due to the matrix’s significantly larger CTE. Additionally, the low-stretched material exhibits an unusually large out-of-plane CTE, exceeding that of the matrix alone, another unexpected result, as the composite was expected to have a lower out-of-plane CTE than the matrix due to the fiber’s significantly smaller transverse CTE. This discrepancy will be discussed further in detail.

### 5.2. Modeling Results

Table 4 presents the results of a simple simulation based on the input data provided in Table 2 and the assumed average volume fractions, without accounting for any fiber misalignment or variation in fiber volume fraction. The results were obtained using Equations (9) and (10), assuming that all the laminates are transversely isotropic.

The first observation is that, for the virgin laminate where the fibers are mostly aligned, there is a significant difference between the measured values and the simulation results. These were expected to be in good agreement, given that the fibers are well-aligned and uniformly distributed in the carbon fiber UD composites with a specific volume fraction. The initial thought that comes to mind is that the fiber CTE properties, especially in the axial direction, might differ from the values in Table 2. It is worth noting that, for the calculated axial CTE of the virgin laminate to fall within the range of the measured values, the axial CTE of the carbon fiber would need to be approximately 10 times smaller $\left(\approx -5\times 10^{-6}/{}^{\circ}C\right)$ than the value reported in Table 2. Despite very limited actual measurement of carbon fiber CTEs in the literature, such a low value is rarely reported, and the assumed value in Table 2 appears to be more realistic, based on the Toray data sheet for T700S [36], the input parameters reported in Ref. [35], or the input data for the Worldwide Failure Exercise [38]. McCartney [31] also highlighted such discrepancies between modeling and experiments when predicting the axial thermal expansion for UD carbon fiber composites based on the input data for the Worldwide Failure Exercise.

One way to explain such discrepancy is that even in the UD virgin carbon fiber laminates, the fibers are actually slightly misaligned by a few degrees with respect to the axial direction. Even if the fibers are perfectly aligned in UD virgin carbon fiber laminates, when cutting small samples for TMA, it is very likely that fibers are oriented a few degrees from their original direction. For understanding, it is assumed that in the virgin UD laminate samples for TMA, fibers are oriented between three to five degrees with respect to the axial direction. Table 5 shows the calculated axial, transverse, and out-of-plane CTEs for such samples together with the shear coefficient of thermal expansion ${\tilde{\alpha }}_{s}$ (See Equation (25)) that arise due to this small misalignment.

**Figure 6:**
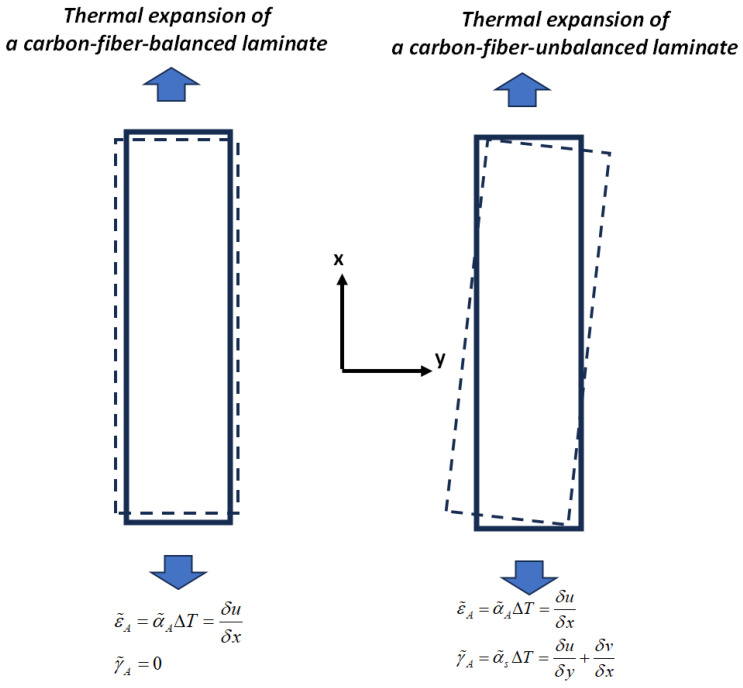
Shear coupling deformation in both balanced and unbalanced laminates under a uniform temperature change ΔT leads to difficulties in characterizing in-plane axial and transverse thermal expansion coefficients due to the non-uniform displacement variation.

It is evident that a small fiber misalignment does not significantly affect the out-of-plane CTE and has only a minimal impact on the transverse CTEs. Its effect on the in-plane axial CTE is larger but cannot explain the large deviation between modeling and measurement. However, it is important to note the resulting value of the shear CTE, which arises due to the unbalanced nature of the laminate caused by the fiber misalignment. The presence of shear thermal expansion coefficients results in a non-uniform axial and transverse displacement change in the experimental sample under a uniform temperature change (see Figure 6). Figure 6 shows the shear coupling deformation in both balanced and unbalanced laminates under a uniform temperature change. The presence of shear CTEs induces shear deformation, which causes variations in axial displacement along the longitudinal direction of the sample and transverse stress in the width direction, all of which affect CTE measurements. Consequently, the calculated values for axial and transverse thermal expansion coefficients may vary depending on the location chosen along the width or length for measuring the displacement change. This effect is more pronounced in carbon fiber composites, where the axial displacement exhibits small negative values that are more susceptible to shear deformation. Such shear deformation can lead to potential mismeasurement of the axial and transverse thermal expansion coefficients if not accounted for during measurement. In extreme cases, the measured values of both in-plane axial and transverse CTEs can deviate by $\pm {\tilde{\alpha }}_{s}$. For the cases considered in Table 5, Table 6 shows the possible range of CET measurements resulting from in-plane shear coupling deformation. The above statement can be an explanation of the very small negative values of the axial CTE for the virgin laminate in Table 3. The significant deviation of the axial CTE in the virgin laminate from theoretical predictions can be attributed to slight fiber misalignment (three to five degrees) in the UD laminate, which induces shear deformation, leading to non-uniform displacement changes and affecting the measured axial CTE.

For the low-stretched and high-stretched carbon staple fiber samples, the fiber orientation distributions are shown in Figure 3 and Figure 4. The laminate analogy formulation described in the previous section is then used to calculate the CTEs. To achieve this, a statistical distribution for fiber volume fractions can be considered for different grains in each tape. In the current work, a normal distribution is assumed for the fiber volume fractions within grains, with the mean value equal to the measured one and a standard deviation of 20%. These values are then applied in Equations (3)–(11) to predict the effective properties for each grain in its local coordinate system. Furthermore, varying thicknesses of the tapes used in sample preparation can also be considered. This is easily incorporated by including thicknesses in Equation (23). Physically, this implies that for a symmetric lay-up, the fiber orientation distribution of the thicker tape has a greater influence on the laminate properties. When considering bending deformations that lead to out-of-plane displacements, additional information about the order of tape placement is necessary, as bending properties depend on variations in material and geometrical properties through the thickness direction. Since no information is available on the specific placement order of each tape, we have limited our modeling to symmetric lay-ups. However, an extension of the model to account for bending deformations is possible [32] if such details become available.

**Figure 7:**
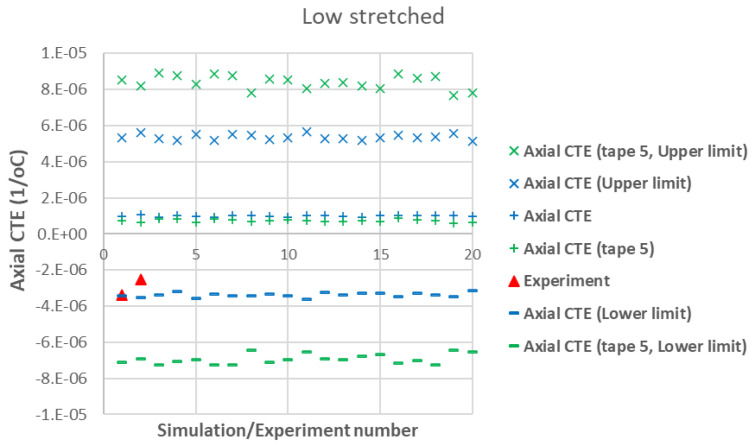
Measured axial CTEs in low-stretched carbon staple fiber samples with average Vf = 39.5% together with simulations with different assumptions.

**Figure 8:**
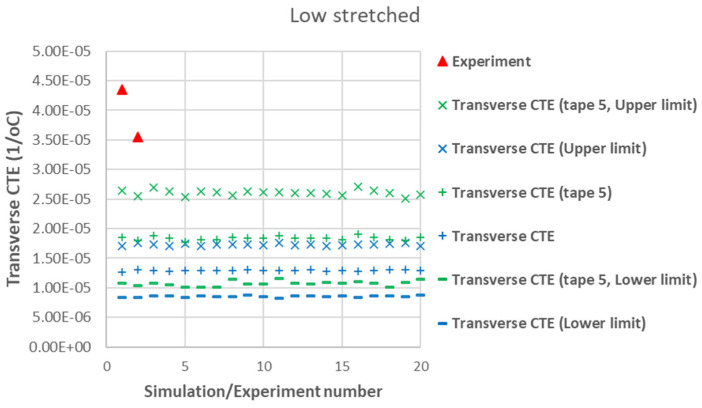
Measured transverse CTEs in low-stretched carbon staple fiber samples with average Vf = 39.5% together with simulations with different assumptions.

**Figure 9:**
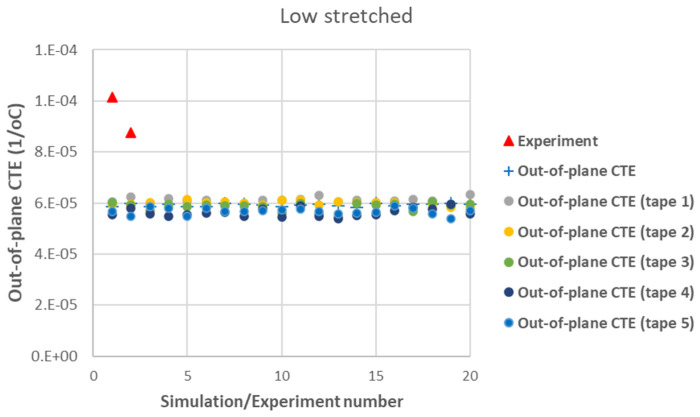
Measured out-of-plane CTEs in low-stretched carbon staple fiber samples with average Vf = 39.5% together with simulations with different assumptions.

Figure 7 presents a comparison between the axial CTE of low-stretched samples obtained experimentally and numerically. Due to the assumption of a statistical distribution for grain fiber volume fractions, multiple simulation results are shown. However, using a normal distribution, the results exhibit little dependency on this variable. It is assumed that all tapes have the same thickness, with their fiber orientation distribution as shown in Figure 3. In addition to the axial CTE, its upper and lower limits ${\tilde{\alpha }}_{A}\pm {\tilde{\alpha }}_{s}$, considering the effects of shear deformation, are also included. To further illustrate the effects of a specific tape’s fiber orientation distribution, the results of a simulation assuming that all plies are made of tape 5 are also presented. It is evident that a tape with a more asymmetric fiber orientation distribution relative to its maximum value leads to greater variation in the measured axial CET due to increased shear deformation under a uniform temperature change. Figure 8 provides a similar comparison for the transverse CTEs of low-stretched samples. Since in-plane shear deformation, caused by shear thermal expansion coefficients, has no effect on out-of-plane deformation under a uniform temperature change, Figure 9 presents only the variation of out-of-plane CTEs. This figure also involves simulations, assuming that all plies are made of a specific tape considering the effects of a specific tape’s fiber orientation distribution.

**Figure 10:**
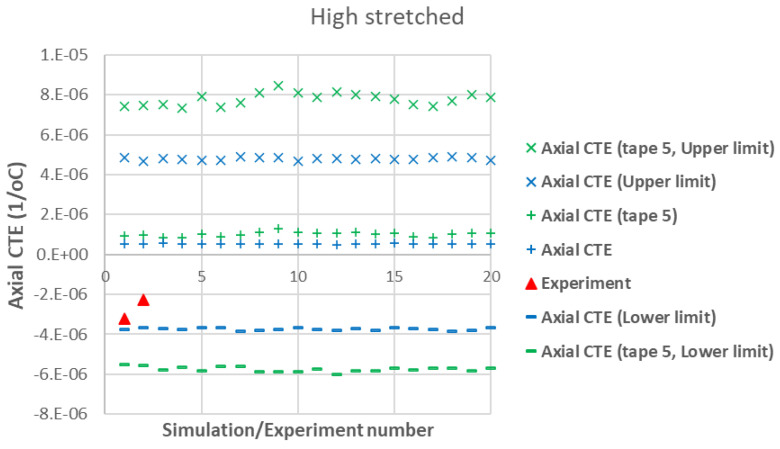
Measured axial CTEs in high-stretched carbon staple fiber samples with average Vf = 46.3% together with simulations with different assumptions.

**Figure 11:**
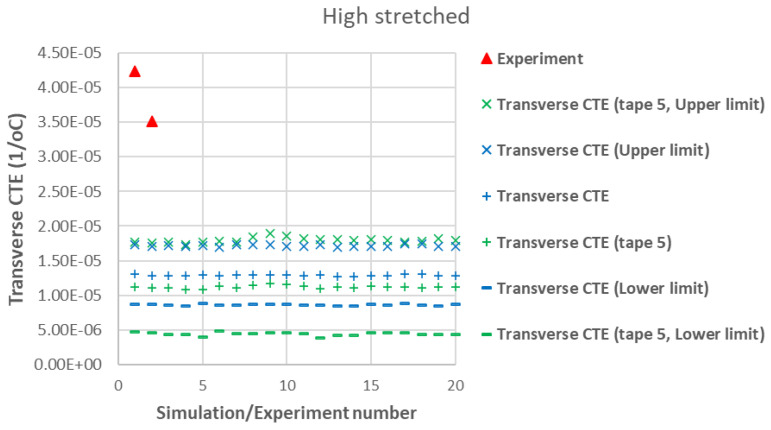
Measured transverse CTEs in high-stretched carbon staple fiber samples with average Vf = 46.3% together with simulations with different assumptions.

**Figure 12:**
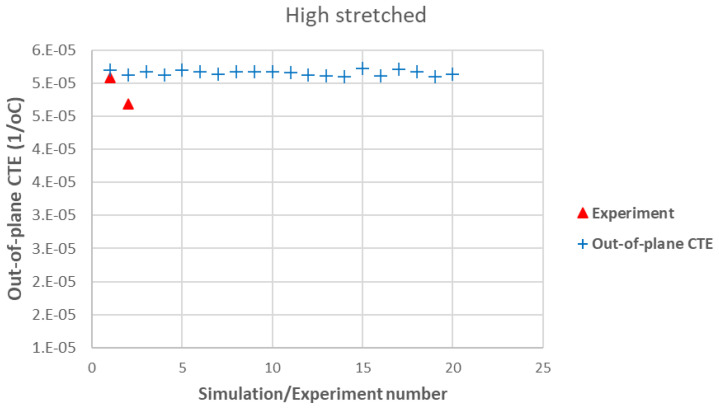
Measured out-of-plane CTEs in high-stretched carbon staple fiber samples with average Vf = 46.3% together with simulations with different assumptions.

Figure 10, Figure 11 and Figure 12 show similar comparisons for high-stretched samples. Since the effect of a specific tape’s fiber orientation distribution on out-of-plane CTEs was small (see Figure 9), it is not shown in Figure 12.

### 5.3. Discussion of the Results

A first observation in Figure 7 and Figure 10 is the good agreement between the lower-bound modeling results and experimental measurements for the axial CTE in both low- and high-stretched samples. This agreement occurs when the effects of all tapes are taken into account in the modeling. The fact that the lower-bound, rather than the actual axial CTE, aligns with the experimental data suggests the presence of shear–tensile coupling in the samples, which significantly influences axial CTE measurements of carbon staple fiber composites. This implies that TMA measurements of axial displacement in carbon staple fiber composites involve additional effects due to shear coupling, but these effects can be excluded through modeling when having information about fiber orientation distributions.

Another observation from Figure 7 and Figure 10 is that the effect of shear CTEs is more pronounced in low-stretched samples. This is evident from the larger difference between the upper and lower bounds in the simulations for low-stretched samples compared to high-stretched samples. Notably, the actual fiber orientation distributions were used to generate these modeling results. This indicates that while changes in processing parameters for high-stretched tapes have not completely eliminated shear coupling, they have reduced it compared to low-stretched samples—both at the tape level (as seen in the simulation for Tape 5) and at the laminate level (when all tapes are considered).

Figure 12 shows a good agreement between the modeling results and experimental data for the out-of-plane CTE in high-stretched samples. In Figure 9 and Figure 12, both experimental and numerical results indicate that the out-of-plane CTEs in carbon staple fiber composites are significantly larger than the transverse CTEs, deviating from the transversely isotropic behavior typically observed in continuous fiber UD composites. This effect is well captured by the developed modeling tool, which incorporates fiber orientation distributions from each tape. There is less agreement between modeling and experiment for out-of-plane CTE in low-stretched samples in Figure 9 while this value is measured to be very large, even larger than the out-of-plane CTE of the matrix alone. This might be due to a greater possibility of out-of-plane bending deformation in low-stretched samples compared to highly stretched ones. Out-of-plane bending deformation occurs, for example, when there is a change in fiber orientations through the thickness of the plate, which happens when there are differences in fiber orientation between individual tapes. The very large out-of-plane CTE in low-stretched samples might be explained by out-of-plane bending deformations, which are not considered in this paper.

The agreement between modeling and experiment for transverse CTEs in Figure 8 and Figure 11 is less strong, possibly due to the assumed input values for the matrix. These values have a greater influence on transverse properties, as they are more dependent on matrix characteristics. As mentioned earlier, the assumed Young’s modulus of 2.76 GPa for the matrix system may be overestimated due to the presence of toughening agents and adhesion promoters, which would also affect the matrix CTE. One potential source of discrepancy in these results is the non-uniform fiber distribution, which may arise from using a closed mold with a relatively small molding tool (170 × 85 mm). This could contribute to additional non-uniformity in fiber orientations beyond the inherent variations in the tapes themselves, which are already considered in the modeling. Additionally, variations in individual tape sections may lead to a non-symmetric laminate with respect to the mid-plane when stacked, potentially introducing curvature after processing or causing in-plane and out-of-plane bending deformations when subjected to thermal loading in the TMA machine. While it is possible to include bending effects in modeling, it requires further information about tape thicknesses and their location through the thickness. Another critical factor is the greater variability observed in the experimental data for transverse CTEs, which could be attributed to these effects. This raises important considerations regarding manufacturing treatments or modeling adjustments to address such inconsistencies, particularly in terms of the re-manufacturability of these samples. In terms of re-manufacturability, it is crucial to ensure that aligned carbon fiber composites maintain consistent quality across different manufacturing cycles. Variability in fiber orientation, misalignment, or changes in fiber quality between batches can impact the thermal and mechanical properties of the composites. Achieving uniformity in these characteristics is vital for ensuring that re-manufactured composites meet the same performance standards as their virgin counterparts. Thus, controlling manufacturing processes and understanding their influence on material properties is essential for maintaining the re-manufacturability and overall reliability of these materials.

While this work focuses on the thermal expansion behavior of carbon staple fiber composites, mechanical properties such as stiffness, damage behavior, interface response, and strength play a crucial role in the performance of engineering structures. The modeling approach developed in this paper can also be applied to predict all elastic and mechanical properties of carbon staple fiber composites.

## 6. Conclusions

This study provides an in-depth experimental characterization and numerical investigation of the coefficients of thermal expansion (CTEs) in highly aligned carbon staple fiber composites. The results demonstrate that fiber alignment, orientation distribution, and volume fraction play important roles in influencing the thermal expansion behavior of these composites. Unlike continuous unidirectional laminates, staple fiber composites exhibit distinct in-plane axial, transverse, and out-of-plane CTE behaviors, which are influenced by fiber misalignment and microstructural variations. Staple fiber composites do not exhibit transversely isotropic behavior. Due to in-plane shear–tensile coupling deformations caused by the asymmetric distribution of fiber orientations, experimental characterization of CTEs requires further considerations.

The experimental findings indicate that the CTE values measured for low-stretched and high-stretched staple fiber composites differ from those predicted by classical micromechanical models, highlighting the need for advanced modeling approaches. The developed laminate analogy approach successfully incorporates detailed fiber orientation distribution, volume fraction variations, and microstructural heterogeneities, leading to more accurate predictions of composite thermo-elastic properties. The results also suggest that unbalanced fiber orientation distributions contribute to in-plane shear–tensile coupling, affecting thermal expansion measurements. For the transverse CTE, the presence of asymmetry in fiber orientation distribution leads to a shear CTE that can alter the measured transverse CTE by approximately 20%, due to the smaller shear CTE compared to the transverse CTE. For the axial CTE, the presence of shear deformation under temperature change can affect the measured transverse CTE by several orders of magnitude, depending on the level of asymmetry in fiber orientation distribution and material properties, due to the significantly larger shear CTE compared to the axial CTE of carbon staple fiber composites.

This study enhances the understanding of the thermo-mechanical behavior of aligned staple fiber composites, paving the way for their optimized design and broader applications in structural and functional components. By improving the efficiency of fiber alignment and reducing material waste, this research supports the sustainability of CFRP production, contributing to efforts aimed at minimizing environmental impact. Future research should extend modeling efforts to investigate the mechanical behavior in terms of stiffness and strength while also improving processing techniques to enhance re-manufacturability.

## Figures and Tables

**Table 1:** Resin mixing method for Hexion system EPIKOTE^TM^ 05545/EPIKURE^TM^ 778/EPIKURE^TM^ 120.

Name	Mixing Ratio (w%)	Mixing Temperature (°C)
EPIKOTE^TM^ Resin 05545	100	80
EPIKURE^TM^ Curing Agent 778	16	25
EPIKURE^TM^ Catalyst 120	3	25

**Table 2:** Fiber and matrix properties.

Material Property	Fiber [35,36] *	Matrix [37] **
Axial Young’s modulus (GPa)	230	2.76
Transverse Young’s modulus (GPa)	15	2.76
Axial Poisson’s ratio	0.25	0.3
Transverse Poisson’s ratio	0.35	0.3
In-plane shear modulus (GPa)	13.7	1.062
Axial CTE (10^−6^/°C)	−0.56	62.5
Transverse CTE (10^−6^/°C)	5.6	62.5

* Carbon fiber is assumed to be transversely isotropic. ** Epoxy matrix is assumed to be isotropic (data obtained from the preliminary data sheet from Hexion on Epikote Resin 05545 published on 2021).

**Table 3:** Experimentally measured thermal expansion coefficients.

Samples	Axial CTE $\left({\tilde{\alpha }}_{A},10^{-6}/{}^{\circ}C\right)$	Transverse CTE $\left({\tilde{\alpha }}_{T},10^{-6}/{}^{\circ}C\right)$	Out-of-Plane CTE $\left({\tilde{\alpha }}_{t},10^{-6}/{}^{\circ}C\right)$
Virgin laminate (Vf = 55%)			
Virgin-1	−4.342	—	—
Virgin-2	−3.169	—	—
Virgin-3	−2.760	—	—
Average	**−3.43**	—	—
Low Stretched (Vf = 39.5%)			
LS-1	−3.356	43.53	101.5
LS-2	−2.515	35.57	87.68
Average	**−2.94**	**39.55**	**94.59**
High Stretched (Vf = 46.3%)			
HS-1	−3.209	42.39	50.80
HS-2	−2.275	35.13	46.84
Average	**−2.74**	**38.76**	**48.82**

**Table 4:** Calculated CTEs without considering the effects of possible fiber misalignment or variation of the microstructure.

Samples	Axial CTE $\left({\tilde{\alpha }}_{A},10^{-6}/{}^{\circ}C\right)$	Transverse CTE $\left({\tilde{\alpha }}_{T},10^{-6}/{}^{\circ}C\right)$	Out-of-Plane CTE $\left({\tilde{\alpha }}_{t},10^{-6}/{}^{\circ}C\right)$
Virgin laminate (Vf = 55%)	0.078	34.95	34.95
Low Stretched (Vf = 39.5%)	0.6124	46.53	46.53
High Stretched (Vf = 46.3%)	0.3356	41.35	41.35

**Table 5:** Calculated CTEs for UD virgin laminate with different fiber orientations (Vf = 55%).

Samples	Axial CTE $\left({\tilde{\alpha }}_{A},10^{-6}/{}^{\circ}C\right)$	Transverse CTE $\left({\tilde{\alpha }}_{T},10^{-6}/{}^{\circ}C\right)$	Out-of-Plane CTE $\left({\tilde{\alpha }}_{t},10^{-6}/{}^{\circ}C\right)$	Shear CTE $\left({\tilde{\alpha }}_{s},10^{-6}/{}^{\circ}C\right)$
$\theta =3^{\circ }$	0.173	34.85	34.95	−3.644
$\theta =4^{\circ }$	0.248	34.78	34.95	−4.853
$\theta =5^{\circ }$	0.343	34.68	34.95	−6.055

**Table 6:** Possible variations in measurements of the axial and transverse CETs for an unbalanced virgin laminate with different fiber orientations (Vf = 55%), corresponding to the case described in Table 5.

Samples	Possible Axial CTE $\left({\tilde{\alpha }}_{A},10^{-6}/{}^{\circ}C\right)$	Possible Transverse CTE $\left({\tilde{\alpha }}_{T},10^{-6}/{}^{\circ}C\right)$
$\theta =3^{\circ }$	0.173 ± 3.644	34.85 ± 3.644
$\theta =4^{\circ }$	0.248 ± 4.853	34.78 ± 4.853
$\theta =5^{\circ }$	0.343 ± 6.055	34.68 ± 6.055

**Figure A1:**
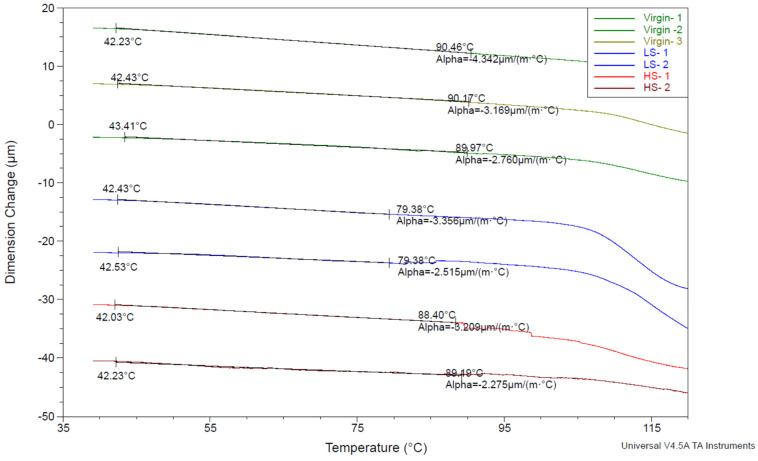
Dimension change in the axial direction versus temperature in low-stretched, high-stretched, and virgin samples.

**Figure A2:**
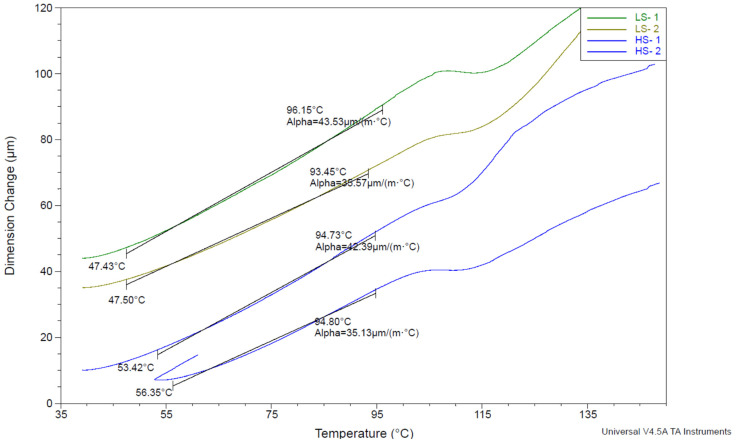
Dimension change in the transverse direction versus temperature in low-stretched and high-stretched samples.

**Figure A3:**
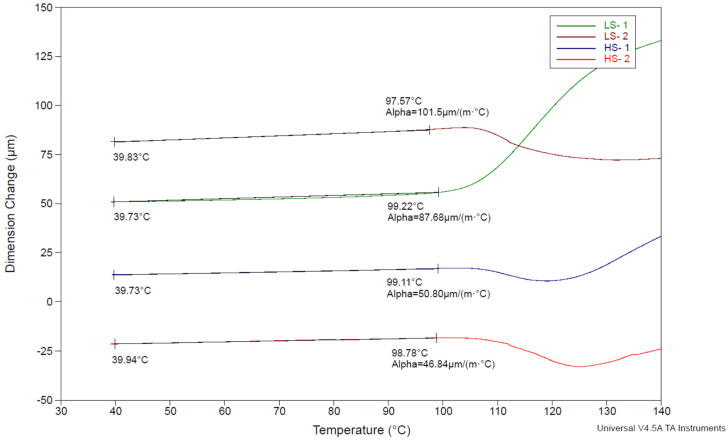
Dimension change in the out-of-plane direction versus temperature in low-stretched and high-stretched samples.

## Data Availability

The data presented in this study are available upon request from the corresponding author due to privacy considerations.

## Appendix A

In this appendix, data obtained from the TMA instrument is provided, showing the variation of sample dimensions with temperature and the temperature range selected for CTE calculation in low-stretched, high-stretched, and virgin laminates.
